# Update on Neuromodulation for Migraine and Other Primary Headache Disorders: Recent Advances and New Indications

**DOI:** 10.1007/s11916-024-01314-7

**Published:** 2025-02-15

**Authors:** Alexandra N. Cocores, Liza Smirnoff, Guy Greco, Ricardo Herrera, Teshamae S. Monteith

**Affiliations:** Department of Neurology─Headache Division, University of Miami, Miller School of Medicine, 1120 NW 14Th Street, 13th floor, Miami, FL 33136 USA

**Keywords:** Neuromodulation, Migraine headache disorder, Primary headache disorder, Non-invasive device, Cluster headache, Non-pharmacologic treatment

## Abstract

**Purpose of Review:**

Neuromodulation techniques currently available for headache management are reviewed in this article, with a focus on recent advances in non-invasive devices for migraine and trigeminal autonomic cephalalgias.

**Recent Findings:**

The currently available FDA-cleared non-invasive devices for migraine include transcutaneous supraorbital and supratrochlear nerve stimulation, single-pulse transcranial magnetic stimulation (sTMS), external concurrent occipital and trigeminal neurostimulation (eCOT-NS), remote electrical neuromodulation (REN), and non-invasive vagal nerve stimulation (nVNS) with indications for migraine and trigeminal autonomic cephalalgias. Emerging non-invasive techniques being explored for use in migraine include transcranial direct current stimulation (tDCS), kinetic oscillation stimulation (KOS), and auricular transcutaneous vagal nerve stimulation (at-VNS). In addition to primary headache, non-invasive neuromodulation is being investigated for comorbid conditions such as depression.

**Summary:**

Non-invasive neuromodulation devices remain a safe, well-tolerated, and effective therapy for patients with primarily migraine and trigeminal autonomic cephalalgias. Ongoing research is needed to determine efficacy in other headache disorders and comorbid conditions.

## Introduction

Primary headache disorders, especially migraine, are common and disabling conditions; in addition to migraine, other disorders such trigeminal autonomic cephalalgias are associated with great unmet therapeutic needs [1, 2]. However, advances in neuromodulation are evolving, resulting in multiple non-pharmacological options. Therapeutic neuromodulation is defined as the alteration of neural pathways, through targeted delivery of electrical, magnetic, or chemical stimulation, to restore function or to relieve symptoms of a neurological basis [3, 4]. Initial work on neuromodulation as therapy for chronic pain began in the late 1960s, with application to the spinal cord and peripheral nerves; in the 1990s, these techniques were increasingly applied to treatment of cephalic neuralgias (including occipital neuralgia) and primary headache disorders [5, 6]. Earlier invasive occipital nerve and deep brain stimulation devices were initially promising[7]; although efficacy was demonstrated in some studies, unacceptable rates of complications have limited integration into clinical practice.

In 2013, the European Headache Federation provided a consensus statement on the clinical use of neuromodulation [8]; the American Headache Society in 2021 provided updates on the integration of neuromodulatory devices into clinical practice [9]; and, the International Headache Society published specific recommendations for optimizing the design and conduct of controlled trials for the acute and preventive treatment of migraine [10].

**Fig. 1:**
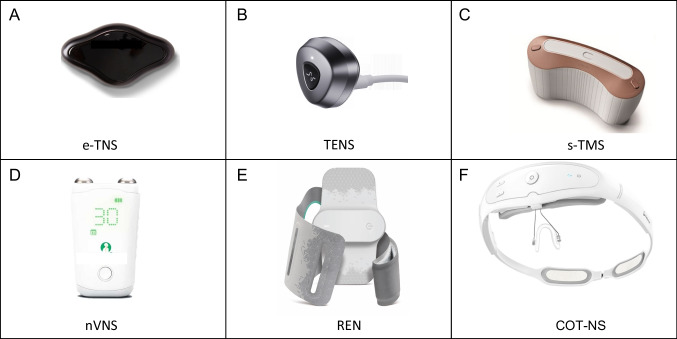
Non-Invasive FDA-Cleared Devices for Primary Headache. The six non-invasive FDA-cleared devices for primary headache, mainly for migraine, include two devices, e-TNS and TENS, using supraorbital and supratrochlear nerve stimulation (A-B), transcranial magnetic stimulation (C) non-invasive vagal nerve stimulation (D), remote electrical neuromodulation (E) and combined trigeminal and occipital neuromodulation (F). Although there are variances in levels of evidence and trial design, all devices are efficacious and have a highly favorable safety profile. Images courtesy of: Cefaly Technology, WAT Medical; electroCore, Theranica Bio-Electronics, Neurolief, and eNeura. Adapted with permission, original source Tepper et al. [31]

Non-invasive neuromodulatory devices designed for headache management are safe and effective alternatives or adjunctive therapies to pharmacologic treatments (Table 1, Fig. 1). They may be advantageous in situations of pharmacological non-response, intolerances, or contraindications (due to medical comorbidities, polypharmacy, pregnancy or the peripartum period) [1]. In this review, we provide an update on the non-invasive neuromodulation devices currently available for primary headache disorders, with a focus on recent advances.

**Table 1:** Comparison of FDA-cleared Non-invasive Devices for Primary Headache Disorders

	**External trigeminal nerve stimulation** (**eTNS)**	**Transcutaneous electrical nerve stimulation (TENS)**	**Single-pulse transcranial magnetic stimulation (sTMS)**	**Noninvasive vagus nerve stimulation (nVNS)**	**Remote electrical neuromodulation (REN)**	**External concurrent occipital & trigeminal nerve stimulation (eCOT-NS)**
**Brand name**	Cefaly, Fig. 1A	HeadaTerm 2, Fig. 1B	Savi Dual, Fig. 1C	GammaCore, Fig. 1D	Nerivio, Fig. 1E	Relivion, Fig. 1F
**Device wearability**	Forehead adhesive	Forehead adhesive	Handheld	Handheld	Armband	Headband
**Electrode Type**	Non-latex adhesive	Conductive silica gel film	External (none)	Conductive gel, non-latex	Non-latex adhesive	Water-based
**Chargeability**	Rechargeable	Rechargeable	Rechargeable	Rechargeable	Non-rechargeable (18-use device)	Rechargeable
**Treatment modality**	Rectangular biphasic compensated impulses with an electrical mean equal to zero, 250 µS impulse width, 60 Hz frequency, maximum intensity of 16 mA with a progressive slope from 1 to 16 mA over 14 min	Pulse repetition frequency of 50 Hz, a pulse width of 125 μs, and an impulse amplitude of 60 V	Single pulse of magnetic stimulation	Electrical signal comprising a 5-kHz sine wave burst lasting for 1 ms (5 sine waves, each lasting 200 μs), with such bursts repeated once every 40 ms (25 Hz), generating a 24-V peak voltage and 60-mA peak output current	Electrical signal comprising a modulated symmetrical biphasic square pulse with a modulated frequency of 100‐120 Hz, pulse width of 400 μs, and output current up to 40 mA	Electrical signal comprising a phase width 100 [micro]s, a pulse frequency 0.33 Hz, trigeminal stimulation intensity up to 5 mA, and occipital stimulation intensity up to 7 mA
**FDA-cleared** **Adult Indications**	Migraine preventive [11] and acute[12, 13]	Migraine preventive [14]	Migraine preventive[15] and acute [4]	Migraine preventive and acute [16, 17] eCH and cCH preventive (adjunctive to medication) [18] eCH acute [19] HC and PH treatment [20, 21]	Migraine preventive and acute [22, 24,23]	Migraine acute [25]
**FDA-cleared** **Pediatric Indications**	None	None	Migraine preventive and acute, age 12 +	Migraine preventive and acute, age 12 +	Migraine preventive and acute, age 8 +	None
**Recommendations for use**	To clean forehead, attach electrode and then device. Press button × 1 for acute or × 2 for prevent program, Pause stim at comfortable and noticeable intensity	Place 2 conductive gels on device, and then adhere device to clean forehead. Press button to start. Press button to stop the increase and maintain intensity	Press button to turn on device. Hold device cradled to back of head. Press treatment button to delivery pulse	Apply to neck, use conductive gel before each stim. Maintain to same side during of treatment Aan alternate sides between treatments	Remove electrode film, place to upper outer arm, adjust armband size and warp around arm. Initiate and control treatment via smartphone app	Adjust device to fit head tightly, place 6 electrode pads, spray pads with water and place arms on sides of clean head above ears until magnet ends lick to connect. Initiate and control treatment (may use smartphone app)
**Acute dosing**	Up to 60 min	Device stops after 20 min. May use “as long as symptoms last.” Device functions for 7 + hours	4 sequential pulses; may repeat	2 two-minutes stimulations; may repeat Up to 24 stims / day (Additional treatments may be considered for trigeminal autonomic cephalalgias)	45 min May use PRN on days used for prevention	20–60 min
**Preventive dosing**	20 min daily	N/A	4 pulses twice daily	2 two-minute stimulations twice daily	45 min every other day	N/A
**Side Effects**	Sleepiness Headache Forehead skin redness, allergy Nausea	None listed	Light-headedness Tingling Tinnitus Dizziness	Application site discomfort, irritation, redness, tingling, prickling Muscle twitching and/or contractions Pain in face, head, neck, teeth Dizziness	Warmth Temporary arm/hand numbness Itching Tingling Muscle spasm Pain in the arm, shoulders, neck Rash or redness at site	Scalp numbness Itching Skin irritation Skin redness Unpleasant sensation while in use
**Safety Considerations**	Not evaluated in those who received supraorbital nerve blocks or Botox treatment in the prior 4 months	None listed	Long-term effects of sTMS are unknown	Not evaluated in those with carotid artery atherosclerosis, cervical vagotomy, clinically significant hyper/hypotension or brady/tachycardia Not evaluated in those with metallic device implanted at/near neck (stent, bone plate/screw)	Not evaluated in those with congestive heart failure, severe cardiac or cerebrovascular disease Should be applied over dry, healthy skin with normal physical sensation and without any metallic implants or in proximity to cancer lesions	Not evaluated in those with suspected or diagnosed heart disease or epilepsy
**Pregnancy Considerations**	Not evaluated previously	Not evaluated previously	2 case reports and 1 case series (26 patients) did not show birth defects or developmental complications [26–28]	On review on invasive VNS in 44 pregnancies did not birth defects or developmental complications [29]	Retrospective controlled study of 59 subjects using REN did not show increased risk of birth defects or developmental complications[30]	Not evaluated previously
**Contraindications**	Metallic or electric devices implanted in the head Cardiac pacemaker or implanted or wearable defibrillator Pain of unknown origin	Acute inflammation, hemorrhagic tendency, arrythmia, epilepsy Skin infection, bleeding, allergy Any cranial implant or cardiac PM Craniocerebral injury, recent maxillofacial injury, brain tumor, meningitis, acute stroke	Conductive metal implants in the head and neck	Any active implanted electronic medical device (Ex. PM, hearing aid) Simultaneous use of other portable electronic device (Ex. TENS unit, muscle stimulator)	Uncontrolled epilepsy Any active implanted electrical device (Ex. PM, hearing aid implant)	Metal implants or shrapnel in the head (except dental implants) Recent (< 3 months) brain or facial trauma Skin abrasions on the forehead or occiput at the contact area Implanted neurostimulators or any implanted metallic or electronic device in the head Cardiac pacemaker or an implanted or wearable defibrillator

ECH (episodic cluster headache), cCH (chronic cluster headache), HC (hemicrania continua), PH (paroxysmal hemicrania), PM (pacemaker)

## Non-Invasive Neuromodulation Devices for Headache Disorders

### Transcutaneous Supraorbital and Supratrochlear Nerve Stimulation

There are two devices that target the supraorbital and supratrochlear nerves in the form of transcutaneous electrical nerve stimulation (TENS). The first device is classified as an external trigeminal nerve stimulator (eTNS) and was designed to target the trigeminal nerve, by providing an electrical pulse to stimulate and desensitize peripheral activity. Specifically, the eTNS (Cefaly device), Fig. 1A, has an electrode strip which when affixed to the forehead sends pulses of electricity to the supratrochlear and supraorbital branches of the ophthalmic division of the trigeminal nerve [12]. The device was FDA-cleared for migraine acute and preventive indications. Although the target is peripheral, whole brain BOLD-fMRI suggests that this device has antinociceptive effects on the anterior cingulate cortex when used preventively [32].

The preventive indication was based upon a randomized sham-controlled (PREMICE) trial of 67 participants across five Belgium-based tertiary headache clinics [11]. The primary outcome measures were change in the monthly migraine days and the percent of participants having at least 50% reduction in monthly migraine days between run-in and the third month. After three months of daily use for 20 min, there was a significant reduction in mean monthly migraine days (6.94 vs 4.88; p = 0.023) which was not seen in the sham group (6.54 vs 6.22; p = 0.608). However, the differences between the two groups failed to meet statistical significance. The 50% responder rate was significantly greater in the verum group compared to sham group (38.1% versus 12.1%). In 2019, e-TNS was studied to determine the safety and efficacy of prevention in a Japanese patient population; the study, which was associated with a compliance of 90%, found a significant decrease in the number of migraine days at 12 weeks after treatment, (8.16 vs. 6.84; p = 0.0036) [33]. A recent prospective observation open-label study published in 2023 showed 30% reduction in headache in patients with chronic migraine in 16.5% of patients, an a non-significant marginal improvement was observed in 42%, showing limited efficacy in individuals with a higher migraine burden [34].

The ACME trial, published in 2019 was a double-blinded, randomized sham-controlled study, conducted across three headache centers in the United States which found that after 60 min of treatment, there was a 59% decrease in pain in the verum group when compared with a 30% decrease in pain among the sham group (*p* < 0.0001) [12]. There were minor adverse events of bothersome paresthesia that caused early cessation of treatment and one reported nausea; 6.6% of subjects could not adhere to the study protocol. The TEAM trial was a real-world study of 538 adults, which demonstrated significant effectiveness of external trigeminal nerve stimulation during acute migraine attacks for 2 h pain freedom (2.5.5% vs 18.3%, p = 0.043) and most bothersome symptom (42.3%; p = 0.001) [13].

In 2021, a second FDA cleared TENS device (HeadaTerm), Fig. 1B, was shown to be effective for the acute treatment of migraine [14]. The study comprised of 78 patients who presented to the emergency department with acute migraine headaches. The patients were instructed to rate their pain on a scale of 1 to 100 by Visual Analogue Scale (VAS) before intervention and 2 h after receiving 20 min of stimulation. Half of the 78 patients received the verum device and other half received sham device. After 20 min of stimulation, the individuals who received the verum device experienced an average 65-point decrease in their pain (± 25), whereas those who received sham device experienced an average 9-point decrease (± 2), (*p* < 0.001). However, the device was FDA-cleared for migraine prevention.

### Single-Pulse Transcranial Magnetic Stimulation

The single-transcranial magnetic stimulation (sTMS) device (SAVI dual), Fig. 1C, is FDA-cleared for the abortive [4] and preventive [15] therapy for migraine. Transcranial magnetic stimulation is theorized to modulate cortical spreading depression, brain excitability, neurotransmission, such as GABAergic circuits, and thalamocortical activity, according to animal studies [35, 36]. The device is applied to the scalp at the back of the head, and a single pulse of magnetic stimulation is delivered to the patient in a non-invasive manner within one second. The device can be used for prevention (user delivers four pulses twice a day) as well as abortively (user delivers 3–4 pulses at the onset of a migraine attack).

The use of sTMS for treatment of acute migraine was first validated in a RCT in which a modified intention-to-treat analysis set of patients with migraine with aura were treated with either sTMS (n = 82) or sham device (n = 82). The primary outcome was pain freedom 2 h after the first attack. In the trial, sTMS was superior to sham: 39% (32/82) in the sTMS group had pain freedom at 2 h, compared to 22% (18/82) who underwent sham stimulation (p = 0.0179).

In the ESPOUSE study, the use of sTMS for prevention of migraine was studied in a multicenter prospective observational study in which patients with migraine disorder were tracked with a headache diary for a 1-month baseline followed by 3 months [15]. The primary outcome was mean reduction in headache days compared to baseline over a 28-day period during weeks 9 to 12. The treatment group used the sTMS device in a preventive fashion (four pulses twice daily) as well as for acute treatment (three pulses up to three times a day for each attack). The sTMS device group reported a 2.75 mean reduction in headache days compared to a statistically derived placebo estimate (performance goal) of 0.63 day mean reduction (*p* < 0.0001). The study also highlights the safety of this device, with no serious adverse events and 29% of patients reporting any adverse events. The most common adverse effects were lightheadedness (3.7%), tingling (3.2%), and tinnitus (3.2%). The main limitation of this study was the lack of a control/sham group. Another limitation is that this study largely sampled patients with episodic migraine (90% of the study) compared to chronic migraine. However, an open label preventive study of 153 patients with high frequency and chronic migraine estimated an overall median reduction in monthly headache days at 3 months was 5.0 days, from 18.0 (IQR: 12.0–26.0) to 13.0 days (IQR: 5.75–24.0) (p = 0.002, r =—0.29)[37]. In a small, open label, feasibility study of adolescents aged 12–17 years, sTMS was found to be feasible, acceptable, and well tolerated when used preventively for a month [38]. Although the sample size was small, there was a significant reduction in mean headache days; and, there were no serious adverse events [38].

### Non-Invasive Vagus Nerve Stimulator

Non-invasive vagus nerve stimulator (nVNS), the Gammacore device, Fig. 1D, is a handheld portable device applied to the neck provides transcutaneous electrical current to stimulate the cervical branch of the tenth cranial nerve. Interest in vagal nerve stimulation for headache disorders emerged after individuals receiving implanted vagal nerve stimulators for epilepsy reported improvement in headache [39]. Proposed mechanisms for efficacy in headache disorders include modulation of the autonomic nervous system, inhibition of cortical spreading depression, and alteration of nociceptive trigeminovascular neurotransmission [40]. A fMRI study to investigate the trigeminal autonomic reflex further elucidated the functional connectivity involved in the modulatory effect of nVNS (via a complex network including the hypothalamus and the left pontine nucleus and the parahippocampal gyrus and the spinal trigeminal nuclei) [41]. In a randomized, crossover, sham-controlled study, vagal nerve stimulation was shown to modulate the nociceptive withdrawal reflex in healthy subjects, which suggests that nVNS may work in part by modulation of the central descending pathways for pain control [42]. Since development, the nVNS device is now FDA-cleared for multiple indications.

The PRESTO (double-blind, sham-controlled RCT) study results led to its clearance for use in adults for the acute treatment of episodic migraine headache: nVNS was superior to sham for pain freedom at 30 min (12.7% vs 4.2%; *p* = 0.012) and 60 min (21.0% vs 10.0%; *p* = 0.023) but not at 120 min (30.4% vs 19.7%; p = 0.067) after the first treated attack (primary endpoint) [16]. For the prevention of episodic migraine headache (PREMIUM; double-blind, sham-controlled RCT), the primary outcome of mean reduction in number of migraine days from the 4-week run-in period (baseline) to the last 4 weeks of the 12-week double-blind period was not met; however, a post hoc analysis of high frequency users was positive for reduction in migraine days (2.27 vs. 1.53; *p* = 0.043) and therapeutic gains were higher in patients with aura (nVNS, − 2.83 days; sham, − 1.41 days; *p* = 0.061) than in those without aura (nVNS, − 2.22 days; sham, − 1.71 days; *p* = 0.15) [17]. The subsequent multicenter, randomized, double-blind, sham-controlled PREMIUM II trial again aimed to evaluate the safety and efficacy of nVNS for migraine prevention; the COVID-19 pandemic led to early termination and the study population was smaller than that needed for full power. The primary endpoint of mean reduction in monthly migraine days was 3.12 for the active group and 2.29 days for the sham group (difference, −0.83; *p* = 0.2329). Percentage of patients with a ≥ 50% reduction in migraine days was greater in the active group (44.87%) than the sham group (26.81%; *p* = 0.0481). Prespecified subgroup analysis suggested that participants with aura responded preferentially. There were no serious device-related adverse events reported [43].

The device is also cleared for prevention of migraine in adolescent patients aged 12 to 17 years: in a preliminary study, 46.8% of the treated migraine attacks (22/47) had a positive response and did not require any rescue medication [44]. Moreover, there is reduced utilization and overall costs in acute migraine management according to cost-effectiveness analyses comparing nVNS plus standard of care compared to standard of care alone [45].

The ACT1 and ACT2 trials (double-blind, randomized sham-controlled trials) studied its use in adults for the acute treatment of episodic cluster headache). Primary endpoints were the response rate, defined as pain relief at 15 min for ACT1 [46] and the proportion of attacks that reached pain-freedom within 15 min for ACT2 [47]; nVNS was superior to sham in episodic but not chronic cluster headache (both endpoints *p* < 0.01) [19]. The use of nVNS for prevention of chronic cluster headache was studied in an open-label, standard-of-care-controlled (PREVA): a significantly higher ≥ 50% response rate during the randomized phase was observed in the SoC plus nVNS group (40% (18/45)) than in the control group (8.3% (4/48)) (*p* < 0.001) [18]. The efficacy and safety of nVNS allows for a unique non-invasive option for cluster headache. In comparison, there is data to support the efficacy of deep brain stimulation, although limited given risk for complications [48]. An RCT with sphenopalatine ganglion stimulation, through surgical implantation, has shown favorable results for chronic cluster headache, although this device is not currently available [49]. Recent real-world studies of nVNS have confirmed findings on efficacy and improvement in quality of life in patients with cluster headache who did not respond to or were intolerant to multiple preventive and/or acute treatments [50, 51]. Lower cost and higher benefits in terms of quality-adjusted life years was also reported in the treatment of chronic cluster headache [52].

Newer explored uses of nVNS include acute treatment of vestibular migraine. A retrospective single-center case series has demonstrated relief of vertigo, headache, and nystagmus [53, 54], which may be attributed to connections between the trigeminal, vestibular, and vagal systems in the brainstem [55]. There is also evidence for use as mini-prophylaxis in menstrual migraine (open label study) [56], indomethacin-sensitive headaches (hemicrania continua and paroxysmal hemicrania) (case series) [20, 21], and primary cough headache (case report) [57]; two patients with short-lasting unilateral neuralgiform headache attacks and cranial autonomic symptoms (SUNA) did not have a significant response to treatment with the device (case series) [58].

### Remote Electrical Neuromodulation

The Remote Electrical Neuromodulation (REN), the Nerivio device, Fig. 1E, was first cleared by the FDA in 2019 for the acute management of migraine. The REN comes as a small device with an armband that fastens around the upper arm. Electrical stimulation, controlled by a smart phone, stimulates upper arm peripheral nerves, and is thought to induce conditioned pain modulation (CPM) in the brainstem which in turn causes serotonergic and noradrenergic modulation resulting in pain inhibition as well as a reduction in associated symptoms [22].

An initial pilot RCT completed in 2017 compared the efficacy of a 20-min of acute REN treatment (at various preset intensities) to sham treatment in 71 patients with episodic migraine who completed a total of 299 treatments. The primary outcome was 50% improvement at 2 h in at least 50% of completed treatments; the trial demonstrated a 46–48% reduction in the two strongest stimulations P200 and P150 vs. 26% improvement with sham stimulation [59]. A follow up double blinded, sham controlled RCT completed in 2019 randomized 126 participants to the treatment group, and 126 participants to the sham group [22]. In this study participants received a 45-min treatment, with intensity controlled by the participant via a smartphone app versus the sham device which was intended to be perceptible but not nociceptive both cases. Patients recorded both levels of pain as well as nausea, photophobia, and phonophobia at the time of treatment, selected their most bothersome symptom (MBS), and reported at 2 h if they had improvement in their MBS. Pain reduction was achieved in 66.7% participants in the active group vs. 38.8% in sham, and pain freedom in 37.4% vs 18.4%.

Another prospective RCT published in 2023 evaluated REN for the preventive treatment of episodic and chronic migraine [23]. The 48-h time interval between treatments, which was based on the REN acute open label study, which demonstrated pain relief at 24 h in 45% of participants in at least 50% of attacks [24]. The 179 participants who completed the study were randomized to complete 45 min treatments of REN or sham treatment every other day for 8 weeks with efficacy measured as reduction in mean monthly migraine days. According to further analysis, results were significant for the episodic (−3.2 ± 3.4 vs. −1.0 ± 3.6, p = 0.003) and chronic (−4.7 ± 4.4 vs. −1.6 ± 4.4, p = 0.001) migraine subgroups separately. Further study into the preventive use of REN device at daily intervals is warranted.

Several studies have been conducted and support additional indications in the pediatric and adolescent age group. A follow up prospective, open label study published in 2021 enrolled patients 12–17 years of age and evaluated primarily the safety of the device use (using the now standardized device settings from the 2019 study), with secondary endpoints including pain relief and pain freedom at 2 h [60]. Of 39 participants who completed the study with at least one test treatment, and at least one treatment, one adverse event (2%) of arm pain was reported. In addition, 35% of participants reported pain freedom at 2 h and 69% reported functional improvement at 2 h. In a preventive real-world study to assess change in monthly migraine treatment days in 83 high frequency adolescent users, results showed a considerable month-to-month reduction in the mean (± SD) number of REN treatment days [61]. These studies were limited due to lack of blinding or a control group using a sham device, but overall demonstrated safety and tolerability of the device for adolescent use.

In addition to its FDA cleared indications for acute and preventive treatment for migraine in those at least 8 years of age, a recent survey based retrospective study published in 2023 evaluated the safety of REN for acute management of migraine in pregnant women [30]; 140 participants including women with migraine using REN during pregnancy and a control group of women with migraine not using REN, there was no statistical difference between gestational age, newborn weight, miscarriage rate, preterm birth rate, birth defect rate, stillbirth rate, or milestones at three months of age.

### External Concurrent Occipital and Trigeminal Neurostimulation

The external concurrent occipital and trigeminal neurostimulation (eCOT-NS), Relivion, Fig. 1F, is an external peripheral nerve stimulation device that combines occipital and trigeminal stimulation for the management of episodic and chronic migraine [25]. The device has an abortive option for acute attacks with neurostimulation lasting 60 min. Stimulating electrodes are arranged in a ring around the patient’s head with a magnetic clasp securing the ring posteriorly. Six electrodes are situated over tissue overlying the supraorbital and supratrochlear branches of the trigeminal nerves anteriorly and over the greater occipital nerve branches posteriorly, with electrodes conducting with the scalp through water which is applied to small inset sponges overlying the electrodes. The pathophysiology underlying the device’s function relies on the trigeminal and cortical neurotransmission to the trigeminocervical complex [62].

The abortive use of the device was validated in a randomized controlled trial utilizing a sham device which utilized a phase width 100 [micro]s, a pulse frequency 0.33 Hz, trigeminal stimulation intensity up to 5 mA, and occipital stimulation intensity up to 7 mA. Participants with chronic and episodic migraine were stratified by gender and included 27 participants in the eCOT-NS group and 28 in the sham group [62]. Subjects were instructed to initiate treatment within 45 ± 15 min of perceived pain prior to treatment recorded on a VAS and again at 1 h, 2 h, and 24 h after treatment initiation. The primary endpoint was the relative change in mean baseline VAS pain score 1-h post-treatment initiation. In the intention to treat population, a significant difference in pain severity (53% vs. 10% in sham group; p = 0.0002) was shown as well as for 2-h post treatment (52% vs. 17.3%; p = 0.034) and 24-h (71.3% vs 34.3%; p = 0.022).

Limitations of this study included small sample size, with chronic migraine largely underrepresented (7.3%). For sustained headache relief (improvement from moderate or severe baseline pain to mild or no pain) was not demonstrated at 24 h. Overall, adverse events were mostly local, with one subject possibly experiencing a device associated headache. There are currently no published data regarding use in headache prevention. A preventive option is available for 20 min once daily, and a prospective, non-randomized, single arm, multi-center study is planned [63].

### Emerging Non-Invasive Techniques

There are several other non-invasive techniques being explored for use in migraine. The use of transcranial direct current stimulation (tDCS) has been studied experimentally. In a recent systematic review and meta-analysis of RCTs for migraine treatment, the authors reported on 11 RCTs that evaluated the efficacy and safety of the primarily anodal but also cathodal stimulation, targeting multiple brain regions, including the primary motor cortex (M1), primary sensory cortex (S1), dorsolateral prefrontal cortex (DLPFC), and visual cortex (VC). They found the potential to reduce the number of migraine days per month with M1 and VC action as well as with VC inhibition [64]. In another systematic review that included 12 studies using mostly anodal tDCS, only 1 out the 12 studies were graded as high quality with low risk of bias [65]. Moreover, the efficacy of combination studies using pharmacological agents and neuromodulation is an area of needed investigation. The TACTIC trial (NCT05161871) will investigate the efficacy of tDCS and monoclonal antibodies acting on calcitonin gene-related peptide as a combined treatment for migraine [66].

Kinetic oscillation stimulation (KOS) has been investigated for acute treatment of migraine. KOS when administered to the nasal cavity, is thought to stimulate the mucosa, and possibly activate the sensory nerve terminals with afferents in the trigeminal nerve. The device consists of an inflatable tip that provides targeted stimulation through a minimally invasive oscillating balloon catheter. In a double-blinded parallel design study randomized 1:1 (active n = 18, placebo n = 17) using a placebo module, comparing active to placebo treatment [67], the study met the primary endpoint, which was change in average pain score (0–10 VAS) when comparing 15 minutes after treatment to pre-treatment baseline; the difference in pain scores between the 2 treatments was 3.3 points (95% confidence interval: 2.3, 4.4), *p* < .001. The difference was significant as early as at 5 minutes into the treatment as well as 2 hours post-treatment. In terms of adverse events, there was one vasovagal event in the placebo group. 

In a recent randomized, double-blind, sham-controlled, multicenter clinical trial, the efficacy of weekly KOS was investigated for the preventive treatment of chronic migraine [68]. For the primary outcome measure, the mean change in monthly headache days with moderate to severe intensity during the assessment period (days 14–42 of treatment) was met. Specifically, the active treatment group had significantly reduced monthly headache days, with moderate to severe intensity, (−3.5 days, n = 67) compared with sham (−1.2 days, n = 65) (p = 0.0132). Treatment-emergent adverse events occurred in 61.8% of participants, which was similar across treatment groups. Nasopharyngitis, dizziness, and epistaxis were most common, occurring in 8.3%, 6.3%, and 6.3% respectively. There were no treatment-related serious adverse events observed.

In a resting state fMRI study comparing 10 patients with acute treatment of migraine attacks and 10 controls during KOS treatment, all patients with migraine responded to KOS treatment within 10-20 minutes [69]. The resting state fMRI provided some initial evidence that KOS may alter intrinsic functional activity of the migraine brain, by targeting the trigeminal parasympathetic reflex implicated in migraine pathophysiology [70]. In a cohort of patients with cluster headache, KOS was shown to target the parasympathetic symptoms, by producing ipsilateral cranial autonomic symptoms, quantified by lacrimation, but not cluster headache attacks [71]. The mechanisms of action are yet to be fully elucidated.

The auricular transcutaneous vagal nerve stimulation (at-VNS) of the left ear sensory vagal area was investigated to assess the efficacy and safety in the treatment of chronic migraine in a single center randomized clinical trial. Specifically, physiological studies have shown that the left vagus nerve has modulatory effects on headache as well as somatic pain [72]. Of the 40 patients that completed the protocol, the 1 Hz group had a significantly larger decrease in headache days per 28 days when compared to baseline than patients in the 25 Hz group (−7.0 ± 4.6 vs. −3.3 ± 5.4 days, p = 0.035) [73].

### Other Indications Relevant to Headache

The efficacy of neuromodulation has also been demonstrated for several related conditions. Invasive vagal nerve stimulation has demonstrated efficacy in the treatment of refractory epilepsy and treatment-resistant depression [74]. There has been evidence indicating its impact on pain receptors in animal models, which has resulted in its growing utilization for the management of various chronic pain syndromes [75] [76]. More recently, nVNS has been shown to improve symptoms and gastric emptying in patients with gastroparesis [77]; additional investigations target acute subarachnoid hemorrhage (VANQUISH, NCT04126408), and acute ischemic stroke (NOVIS, NCT04050501).

Research has demonstrated that individuals suffering from chronic migraine are at a heightened risk of developing major depressive disorder over the course of their illness, ranging from 2 to 4 times increased risk [78]. Therefore, the availability of treatment modalities that can effectively address both disorders is highly advantageous. A protocol using prefrontal transcranial magnetic stimulation has been shown to be a safe and effective treatment for treatment resistant major depressive disorder [79]. In addition, nVNS has been shown to enhance cognitive emotional regulation in patients with major depressive disorder [80]. A study using eCOT-NS is actively recruiting to investigate the device’s utility in major depressive disorder (MOOD, NCT04279522 [81]. In 2022, the FDA granted breakthrough device designation for the gammaCore device to be used in the treatment of posttraumatic stress disorder in military personnel [82].

## Conclusion

There have been rapid developments in the use of non-invasive neuromodulation for primary headache disorders. Peripheral neurostimulation is an effective and safe way to modulate the central nervous system in headache practice. Although there has been wide variability in study design and in each device’s ability to achieve key endpoints, these neuromodulation devices have wide applicability [83].

Despite their efficacy and safety, limitations of use remain. With only a few very recent exceptions, devices are generally considered investigational or experimental and are not covered by conventional insurance plans. Studies are needed to assess cost effectiveness, which may improve providers’ willingness to recommend neuromodulation and improve overall patient access [84]. Going forward, research on neuromodulation for the potential treatment of other primary headaches, in addition to secondary headaches such as medication overuse headache and post-traumatic headache is warranted. Lastly, better methods are needed to optimize clinical trials efforts and to translate evidence into clinical practice.

## Data Availability

No datasets were generated or analysed during the current study.
